# War Medicine

**Published:** 1918-08

**Authors:** 


### WAR MEDICINE

The American Red Cross in France by founding the Research Society, in cooperation with the American Expeditionary Forces, has established a high grade medical school for army medical officers — that is, if Dr. Senn was correct in his estimate of the functions of medical societies. In the *Medical Bulletin* the Red Cross has further supplied the American Army surgeons with a "text-book" on the medicine, surgery, and hygiene of the war, which, if studied, and the knowledge contained in it applied, will aid materially in reducing the non-effective rate from wounds and illness among our soldiers.

Major Alexander Lambert, Medical Advisor of the American Red Cross in France, in announcing the organization of the Research Society and the establishment of the *Medical Bulletin*, stated their aims as follows[1]: "First, to encourage periodic meetings of the men concerned; second, to make available the reports of the latest methods of treatment for war injuries and diseases, by means of a publication. In order to provide for meetings it seems advisable to found a Research Society to meet once a month in Paris, thus per-

1. *Medical Bulletin*, Vol. I, No. 1, November, 1917.

mitting men engaged in scientific investigation to exchange views. To review the medical literature, a journal has been started: it will contain abstracts of papers read at the monthly meetings of the society; and also of articles appearing in the French, English, and American journals. It will be issued monthly, and will be available for the physicians and surgeons with the American Army in France, and for those of our Allies who may find it helpful."

With the hope of increasing the scope and usefulness of its medical publication, the Red Cross, through the courtesy of Brigadier-General Ireland, Chief Surgeon of the American Expeditionary Forces, has secured the services of Major Seale Harris, M.R.C., as Editor-in-Chief. Major Harris, acting also as Secretary of the Research Society, will thus relieve Major Kenneth Taylor, M. R. C., whose faithful and indefatigable services have made possible the success of both the Research Society and the *Medical Bulletin*; and so enable Major Taylor to devote his time exclusively to the important research work which is being done under his direction at the Goelet Fund and Red Cross Laboratories. Major Taylor, however, has been made Honorary Secretary, and remains as an active member of the Research Committee. Dr. E. Bradlee Watson, who has acted as Managing Editor of the *Medical Bulletin* from the start, will continue to serve in that capacity. The endeavor will be made to render the *Bulletin* of maximum usefulness to the rapidly increasing Medical Corps of the American Expeditionary Forces.

There can be no question but that the funds spent by the Red Cross in publishing the *Medical Bulletin* have been wisely invested, because it is fulfilling a distinctive mission in a field that needs to be covered; besides, it is the only medical periodical published for the American Expeditionary Forces. This medical monthly of the Red Cross is not a medical journal in a strict sense, because it does not publish articles, and many other features of a medical journal are lacking in its pages. It has, however, one function of a journal, in that it makes the effort to disseminate recent medical knowledge to a large number of physicians.

It has been thought by some that the title *Medical Bulletin*

does not fittingly describe the functions of this publication and since the words " War Medicine, Surgery, and Hygiene " — originally carried as a sub-title on the front page of the *Medical Bulletin* — seemed to cover the field intended for the monthly medical publication of the Red Cross, it was decided to change the name; and the last number was published as *War Medicine, Surgery, and Hygiene*. After one issue it was further suggested that since the publication will be called by the short title, *War Medicine*; and since the word " medicine " really embraces every field of endeavor in which the American physicians in France are engaged, it has been decided to drop the words " Surgery and Hygiene ". Therefore *War Medicine* will be the title beginning with this number.

It will also be observed that in this number there are 144 pages instead of 64 as in former issues, and a department of Editorial Comments has been added. Circulars and bulletins of interest to medical officers, because they are sent out by the Chief Surgeon of the American Expeditionary Forces, will also be published in *War Medicine*, and other improvements are contemplated; but in general, the policy will be adhered to which, from the first number, made the *Medical Bulletin* a marked success.

Since it publishes resumés, and sometimes the entire text, of papers and addresses that are presented before the Research Society, *War Medicine* carries a message of very great importance to the thousands of American physicians who are beginning to have actual experience in war surgery, and who are dealing with the problems in medicine and hygiene that are incident to the movement of large bodies of troops. There have been regrettable delays in getting this valuable material published and distributed. It is hoped, however, that when the services of a medical reporter, who has been cabled for, have been secured, the proceedings of the Research Society may be published and therefore be made available to medical officers within three or four weeks after the meetings.

The prompt publication of anything, however, in a country at war is extremely difficult. The printing industry in France has suffered as much as have many others — publishers say more, because type-setters and other experienced skilled laborers are very scarce. The staff of *War Medicine* at this time is limited, and there have been delays on account of the censorship. There have also been difficulties in the distribution of the *Medical Bulletin* since the space on a railway train required to carry a sack of mail can be utilized to transport a soldier. But all of these problems are being attacked and it is hoped that after one or two more issues, *War Medicine* may be published promptly in the early part of each month.

The American Red Cross in France desires every medical officer in the American Expeditionary Forces to receive a copy of *War Medicine*; a large number of copies are sent to the Surgeon General's office in Washington for the medical officers in training in the United States; and a liberal supply is distributed to the medical officers of the Allied Armies. *War Medicine*, therefore, has a wide distribution and it hopes to disseminate medical knowledge which, if applied, will reduce the non-effective rates in our army, thus helping to win the war.

"To err is human", and errors in policy and in the typographical work of *War Medicine* will surely occur; but the effort will be made to make as few mistakes as possible, and to profit by them when they occur. Criticism is invited, and suggestions offered to make *War Medicine* more helpful to medical officers will be appreciated. It is published with the idea of serving the American physicians who have made sacrifices in order to perform a patriotic duty for their country; and if it can be regarded as one of the up-to-date "textbooks" read by them, and the knowledge thus acquired be used to increase the efficiency of the Medical Department of the American Expeditionary Forces, the mission of the medical publication of the American Red Cross in France will have been fulfilled.

